# Supplementary material for: Trichoderma harzianum Volatile Organic Compounds Regulated by the THCTF1 Transcription Factor Are Involved in Antifungal Activity and Beneficial Plant Responses
Source: J Fungi (Basel). 2023 Jun 11;9(6):654. doi: 10.3390/jof9060654 (PMC10302578; doi:10.3390/jof9060654)
Supplement: Supplementary file 1 [file jof-09-00654-s001.zip › Table S1.pdf]

Table S1. Oligonucleotides used for qPCR analysis of *Trichoderma* and *Arabidopsis* genes.

Organism	Name	Sequences (5′ → 3′)	Gene encoding	References
Trichoderma	LAE-fw2	GCCATGAGCATGATGCCTATG	Putative methyltransfersase (LAE1)	This work
	LAE-rev2	AGTAGGCGTGGTAGCGCAGAA		This work
	5468-fw	GACGTCGTCGGATTGGACTAT	Putative thiopurine S-methyltransfersase	This work
	5468-rev	GCCCTCCTCAATCGACTTGTA		This work
	81579-fw	AGGGCGATGCTAATGCTCTCA	Putative SAM-methyltransfersase	This work
	81579-rev	GGATACCAGATGAAGCCTCCA		This work
	506014-fw	AAAAGAGACGAGCTTGTCCGC	Putative SAM-methyltransfersase	This work
	506014-rev	CGCTCTAAGAGTTGTGATGCC		This work
	492690-fw	CTGGCGGCTACCTTGAGTT	Putative prohibitin protein containing a methyltransferase domain	This work
	492690-rev	GCCCAGCCACAGCTTTGTT		This work
	Act-1	ATCGGTATGGGTCAGAAGGA	Actin	[44]
	Act-2	ATGTCAACACGAGCAATGG		[44]
Arabidopsis	PR-1-fw	CAAAGTGAGGTGTAACAATGGTGGA	Pathogenesis-related protein 1 (PR-1)	This work
	PR-1-rev	ATGGCTTCTCGTTCACATAATTCCC		This work
	VSP2-fw	GTTAGGGACCGGAGCATCAA	Vegetative storage protein 2 (VSP2)	This work
	VSP2-rev	TCAATCCCGAGCTCTATGATGTT		This work
	PDF1.5-fw	GGTTGCTCTTGTTCTCTTTGCT	Plant defensin 1.5 (PDF1.5)	This work
	PDF1.5-rev	CTCTGCACTGATAATCGTTTACAC		This work
	Actin-fw	CTCCCGCTATGTATGTCGCC	Actin	[45]
	Actin-rev	TTGGCACAGTGTGAGACACAC		[45]
