# Supplementary material for: Trichoderma harzianum Volatile Organic Compounds Regulated by the THCTF1 Transcription Factor Are Involved in Antifungal Activity and Beneficial Plant Responses
Source: J Fungi (Basel). 2023 Jun 11;9(6):654. doi: 10.3390/jof9060654 (PMC10302578; doi:10.3390/jof9060654)
Supplement: Supplementary file 1 [file jof-09-00654-s001.zip › Table S2.pdf]

Table S2. Putative identification of VOCs identified through PTR-Qi-TOF-MS analysis. (**a)** Compound rank (**b)** Mass to charge ratio measured by the Mass Spectrometer (**c)** Compound's chemical formula (H+ added by protonation) (**d)** Putative identifications (**e)** Compound classification based on their chemical and biochemical properties (**f)** Fungal volatile emission using PTR-ToF-MS citations (**g)** PTR-TOF citations (**h)** Fungal VOCs citations.

Compound (a)	Measured Mass m/z (b)	Protonated chemical formula (c)	Putative identifications (d)	Chemical and functional grouping (e)	Fungal volatile emission using PTR-ToF-MS citations (f)	PTR-TOF citations (g)	Fungal VOCs citations (h)
1	29.040	C2H5+	Ethylene; Ethyl radical (Ethanol fragment)	Hydrocarbon	[48]		
2	31.018	CH3O+	Formaldehyde	Carbonyl compounds	[48]		
3	33.033	CH5O+	Methanol	Alcohols	[48]		
4	39.024	C3H3+	Isoprene fragment	Hydrocarbon		[56,57]	
5	40.026	-	Unknown				
6	41.040	C3H5 +	Alkyl fragment; 2-Methyl-3-buten-2-ol; Acetone fragment	Hydrocarbon		[58,59]	
7	42.009	-	Unknown				
8	42.042	C3H5 +	Alkyl fragment	Hydrocarbon	[49]		
9	43.020	C2H3O+	Acetic acid fragment	Carboxylic acids	[48,50]		
10	43.055	C3H7+	Alkyl fragment	Hydrocarbon		[60]	
11	45.034	C2H5O+	Acetaldehyde	Carbonyl compounds	[48]		
12	47.043	C2H7O+	Ethanol	Alcohols	[48]		
13	49.010	CH5S+	Methanethiol	Sulphur compounds	[48]		
14	49.028	-	Unknown				
15	53.002	C3OH+	Fragment (ester)	Carbonyl compounds		[61]	
16	53.013	-	Unknown				
17	53.039	C4H5+	Cyclobutadiene	Hydrocarbon	[48]		
18	54.033	-	Unknown				
19	55.016	-	Unknown				
20	57.032	C3H5O+	2-Propenal (Acrolein)	Carbonyl compounds	[48]		
21	57.070	C4H9+	1-Butene (Alkyl fragment); Butanol fragment	Hydrocarbon	[48]	[62]	

22	59.051	C3H7O+	Acetone	Carbonyl compounds	[48]		
23	61.026	C2H5O2+	Acetic acid	Carboxylic acids	[48]		
24	63.045	C2H6O2H+	Acetaldehyde-water cluster	Carbonyl compounds		[61]	
25	67.053	C5H7+	3-Penten-1-yne; 2-Pentenal fragment-; 1,3-Cyclopentadiene	Alkenes	[48]	[61,63]	
26	69.033	C4H5O+	Furan	Terpenes	[48]		
27	69.069	C5H9+	2-Methyl-1,3-butadiene (isoprene); Cyclopentene	Alkenes	[48]		
28	71.049	C4H7O+	3-Buten-2-one (Methyl vinyl ketone); Methacrolein	Carbonyl compounds	[48]		
29	74.004	-	Unknown				
30	74.099	-	Unknown				
31	75.027	-	Unknown				
32	75.081	C4H11O+	2-Methyl-1-propanol (isobutanol)	Alcohols	[48]		
33	77.059	-	Unknown				
34	79.038	-	Unknown				
35	79.053	C6H7+	Methanol trimer or Phenyl ion or Benzene	Benzenoids		[60]	
36	79.074	-	Unknown				
37	81.034	C5H5O+	2,4-Cyclopentadiene-1-one	Carbonyl compounds		[63]	
38	81.069	C6H9+	Alkyl fragment (hexenals/hexenols/terpenoids)	Hydrocarbon	[48]		
39	83.048	C5H7O+	2-Methyl furan	Terpenes	[48]	[61]	
40	83.068	-	Unknown				
41	83.083	C6H11+	Hexanal fragment; 2,3-Dimethyl-1,3-butadiene	Alkenes	[51,52]	[61]	
42	85.064	C5H9O+	Pentenone; Cyclopentanone	Carbonyl compounds	[50]		
43	85.100	C6H15O+	Hexanol fragment	Alcohols		[60,61]	
44	87.044	C4H7O2+	Butan-4-olide	Carbonyl compounds	[48]		

45	87.080	C5H11O+	2-Methylbutanal; Pentanal; Pentanone	Carbonyl compounds	[48,52]		
46	89.059	C4H9O2+	Butanoic acid; Ethyl acetate; 3-Hydroxy-2-butanone (Acetoin)	Carboxylic acids/ester	[48]		
47	89.085	-	Unknown				
48	91.056	C7H7+	Xylene fragment	Benzenoids		[56]	
49	91.074	C4H11O2+	2,3-Butanediol	Alcohols	[48]	[61]	
50	92.060	-	Unknown				
51	93.037	C3H9OS+	2-Methyl-mercaptoethanol	Sulfur compounds	[48]		
52	93.069	C7H9+	Monoterpene fragment	Hydrocarbon		[56]	
53	93.090	C7H9+	Methylbenzene (Toluene)	Benzenoids	[52]		
54	95.085	C7H11+	Monoterpene fragment	Terpenes		[57]	
55	97.028	C5H5O2 +	Furfural; 2-Furancarboxaldehyde	Carbonyl compounds	[48]		
56	97.064	C6H9O+	2,4-Dimethylfuran	Terpenes	[48]		
57	97.100	-	Unknown				
58	99.081	C6H11O+	Hexenal isomeres	Carbonyl compounds	[51]		
59	101.059	C5H9O2+	Oxo-pentanal; Pentane-2,3-dione; γ-valerolactone	Carbonyl compounds	[48,50]		
60	101.096	C6H13O+	Hexanal; Hexanone	Carbonyl compounds		[63]	
61	103.076	C5H11O2+	4-Hydroxy-3-methyl-2-butanone	Carbonyl compounds	[48]		
62	105.070	C4H9O3+	4-Hydroxybutanoic acid	Carboxylic acids/ester	[51]		
63	107.049	C7H7O+	Benzaldehyde	Carbonyl compounds	[53]	[61]	
64	107.068	-	Unknown				
65	107.084	C8H11+	Ethylbenzene; Dimethylbenzene (Xylene); Monoterpene fragment	Benzenoids	[48]	[56]	
66	107.105	-	Unknown				
67	109.102	-	Unknown				
68	111.046	C6H7O2+	2-Acetylfuran	Terpenes	[48]		

69	111.080	-	Unknown				
70	111.115	C8H15+	4-Methyl-1,3-heptadiene	Alkenes	[48]		
71	117.090	C6H13O2+	Acetone dimer; Hexanoic acid; Ethylbutanoate	Fatty acid esters	[51]		
72	119.049	C6H15S+	1-(Methylthio) pentane; Benzofuran	Sulfur compounds	[48]		
73	119.085	C9H11+	Indane; Methyl-styrene	Benzenoids		[63]	
74	119.106	-	Unknown				
75	121.068	C5H13OS+	2-Hydroxyethyl propyl sulfide	Sulfur compounds	[48]		
76	121.101	C9H13+	1,2,4-Trimethylbenzene	Benzenoids	[48]		
77	121.121	C9H13+	1-Ethyl-3-methyl-benzene; Propyl-benzene; Monoterpene (*p*-cymene) fragment	Benzenoids	[50]	[56]	
78	123.044	C4H11O2S+	Diethanol sulfide	Sulfur compound	[48]		
79	123.080	C8H11O+	1-Methoxy-3-methylbenzene	Benzenoids	[48]		
80	123.116	C9H15+	Sesquiterpene fragments	Sesquiterpenoids	[54]		
81	127.112	C8H15O+	2-Octenal/1-Octen-3-one	Carbonyl compounds	[51]		
82	129.090	C7H13O2+	5-Propyldihydro-2(3H)-furanone; γ-Heptalactone; 2-Methyl-2E-hexenoic acid; 4-Pentenyl acetate	Carbonyl compounds/Carboxylic acids/ester		[61]	[66]
83	129.127	C8H17O+	Octanal; 1-Octen-3-ol; 3-Octanone	Carbonyl compounds	[51]		
84	131.107	-	Unknown				
85	135.081	C9H11O+	3-Methylacetophenone	Carbonyl compounds		[63]	
86	135.111	C10H15+	*p*-Cymene	Terpenes	[48]		
87	135.137	-	Unknown				
88	137.133	C10H17+	Monoterpenes	Terpenes	[48]		
89	139.075	C8H11O2+	2-Methoxy-4-methylphenol (Creosol)	Alcohols		[63]	[67]

90	139.112	C9H15O+	2-Pentylfuran; Tyrosol	Terpenes	[48]		
91	139.145	-	Unknown				
92	143.106	C8H15O2+	2,3-Octanedione	Carbonyl compounds	[48]		
93	143.144	C9H19O+	Nonanal; Nonanone; 3,5-dimethyl-4-heptanone/2(3H)-Furanone; 5-butyldihydro	Carbonyl compounds	[49]	[61]	
94	145.122	C9H21O+	Dimer of tetrahydrofuran; Octanoic acid	Alcohols	[55]	[64]	
95	147.116	C11H15+	Cyclopentylbenzene	Benzenoids		[57]	
96	147.138	C8H19S+	2-Ethyl-1-hexanethiol	Sulfur compounds	[48]		
97	149.132	C11H17+	1-Ethylpropyl-benzene	Benzenoids	[48]		
98	151.109	-	Unknown				
99	151.148	C6H15S2+	Methyl pentyl disulfide	Sulfur compounds	[48]		
100	155.146	-	Unknown				
101	157.121	-	Unknown				
102	157.158	C10H21O+	Decanal	Carbonyl compounds	[48]		
103	159.138	C9H19O2+	2-Methylbutyl 2-methylpropanoate; 2-Methylbutyl isobutyrate"	Carboxylic acids/ester	[48]		
104	161.134	-	Unknown				
105	163.147	C12H19	Sesquiterpene fragment	Terpenes		[57]	
106	167.056	-	Unknown				
107	167.108	C10H15O2+	6-Pentyl-2*H*-pyran-2-one (6PP)	Carbonyl compounds			[68-70]
108	167.144	C11H19O+	2-Heptylfuran	Terpenes	[48]		
109	173.134	-	Unknown				
110	177.161	-	Unknown				
111	181.159	-	Unknown				
112	189.128	-	Unknown				
113	191.145	C13H19O+	(E)-β-Damascenone	Carbonyl compounds		[57]	
114	193.160	C13H21O +	β-Ionone	Carbonyl compounds		[65]	

115	195.175	C13H23O+	6,10-Dimethyl-5,9-undecadien-2-one (Geranylacetone)	Carbonyl compounds	[48]		
116	203.180	C15H23+	Sesquiterpenes (eg. curcumene. calamene) and Terpene fragment	Terpene		[57]	
117	205.197	C15H25+	Sesquiterpenes	Sesquiterpenoides	[48]		
118	207.140	-	Unknown				
119	209.155	-	Unknown				
120	221.151	-	Unknown				
121	221.191	C15H25O+	Caryophyllene oxide	Terpenoids		[57]	[71]
122	223.064	-	Unknown				
123	223.206	C15H27O+	Cedrol; Nerolidol	Sesquiterpene alcohol	[48]		
124	225.046	-	Unknown				
125	227.039	-	Unknown				
126	231.889	-	Unknown				
127	265.856	-	Unknown				
128	266.856	-	Unknown				
129	297.082	-	Unknown				
130	299.061	-	Unknown				
