# Supplementary material for: Trichoderma harzianum Volatile Organic Compounds Regulated by the THCTF1 Transcription Factor Are Involved in Antifungal Activity and Beneficial Plant Responses
Source: J Fungi (Basel). 2023 Jun 11;9(6):654. doi: 10.3390/jof9060654 (PMC10302578; doi:10.3390/jof9060654)
Supplement: Supplementary file 1 [file jof-09-00654-s001.zip › Table S3.pdf]

Table S3. Significantly different VOCs identified according to one-way analysis of variance (ANOVA) followed by Fisher's least significant difference method (Fisher's LSD) analysis ($p < 0.05$) emitted by the three *Trichoderma* strains, at 5 and 9 days after inoculation (T34 = wild type; Dj = ΔJ3-16 ectopic integration transformant; DD = ΔD1-38 disruptant transformant).

**5 days data set**							**9 days data set**							
	***f* value**	***p* value**	**-LOG10(p)**	**FDR**	**Fisher's LSD**			***f* value**	***p* value**	**-LOG10(p)**	**FDR**	**Fisher's LSD**		
m/z 61.026+BB5:H43	1903.1	1.50E-12	11.823	9.5511E-11	DD - Dj	DD - T34	m/z 85.064	1191.9	1.23E-11	1.09E+01	1.38E-09	DD - Dj	DD - T34	
m/z 129.090	474.6	7.54E-10	9.1225	1.9159E-08	DD - Dj	DD - T34	m/z 107.105	1055.1	2.12E-11	1.07E+01	1.38E-09	DD - Dj	DD - T34	
m/z 79.038	214.1	2.58E-08	7.589	4.0899E-07	DD - Dj	DD - T34	m/z 71.049	302.37	5.60E-09	8.25E+00	1.46E-07	DD - Dj	DD - T34	
m/z 101.059	123.04	2.91E-07	6.5359	4.1081E-06	DD - Dj	DD - T34	m/z 95.08	259.04	1.11E-08	7.95E+00	2.41E-07	DD - Dj	DD - T34	
m/z 147.138	19.098	5.77E-04	3.2385	0.0067853	DD - Dj	DD - T34	m/z 29.040	241.35	1.52E-08	7.82E+00	2.82E-07	DD - Dj	DD - T34	
m/z 129.127	18.391	6.62E-04	3.179	0.0067853	DD - Dj	DD - T34	m/z 79.053	109.98	4.73E-07	6.32E+00	6.84E-06	DD - Dj	DD - T34	
m/z 87.080	18.149	6.95E-04	3.1583	0.0067853	DD - Dj	DD - T34	m/z 161.134	99.284	7.36E-07	6.13E+00	9.57E-06	DD - Dj	DD - T34	
m/z 119.049	15.742	1.15E-03	2.9387	0.0079333	DD - Dj	DD - T34	m/z 121.068	19.469	5.38E-04	3.27E+00	5.26E-03	DD - Dj	DD - T34	
m/z 43.02	15.701	1.16E-03	2.9347	0.0079333	DD - Dj	DD - T34	m/z 119.106	19.221	5.64E-04	3.25E+00	5.26E-03	DD - Dj	DD - T34	
m/z 40.026	15.683	1.17E-03	2.933	0.0079333	DD - Dj	DD - T34	m/z 123.080	18.985	5.90E-04	3.23E+00	5.26E-03	DD - Dj	DD - T34	T34 - Dj
m/z 39.024	15.475	1.22E-03	2.9128	0.0079333	DD - Dj	DD - T34	m/z 137.133	18.473	6.52E-04	3.19E+00	5.26E-03	DD - Dj	DD - T34	
m/z 59.051	14.926	1.39E-03	2.8583	0.0079333	DD - Dj	DD - T34	m/z 123.044	18.178	6.91E-04	3.16E+00	5.26E-03	DD - Dj	DD - T34	
m/z 85.064	14.774	1.44E-03	2.8429	0.0079333	DD - Dj	DD - T34	m/z 42.009	17.911	7.28E-04	3.14E+00	5.26E-03	DD - Dj	DD - T34	
m/z 127.112	12.209	2.73E-03	2.5639	0.011955	DD - Dj	DD - T34	m/z 147.138	17.909	7.29E-04	3.14E+00	5.26E-03	DD - Dj	DD - T34	
m/z 266.856	12.053	2.85E-03	2.5455	0.012054	DD - Dj	DD - T34	m/z 151.148	16.668	9.42E-04	3.03E+00	5.65E-03	DD - Dj	DD - T34	
m/z 265.856	11.26	3.55E-03	2.4496	0.014096	DD - Dj	DD - T34	m/z 101.059	16.596	9.56E-04	3.02E+00	5.65E-03	DD - Dj	DD - T34	
m/z 77.059	9.3493	6.35E-03	2.197	0.02069	DD - Dj	DD - T34	m/z 147.116	14.093	1.69E-03	2.77E+00	8.93E-03	DD - Dj	DD - T34	
m/z 107.068	9.197	6.68E-03	2.1754	0.021202	DD - Dj	DD - T34	m/z 89.059	13.929	1.76E-03	2.76E+00	8.93E-03	DD - Dj	DD - T34	
m/z 145.122	8.6534	8.01E-03	2.0962	0.02482	DD - Dj	DD - T34	m/z 107.049	13.501	1.95E-03	2.71E+00	9.07E-03	DD - Dj	DD - T34	
m/z 159.138	7.1706	1.37E-02	1.8625	0.041505	DD - Dj	DD - T34	m/z 75.081	13.241	2.08E-03	2.68E+00	9.35E-03	DD - Dj	DD - T34	
m/z 54.033	6.8883	1.53E-02	1.8146	0.045261	DD - Dj	DD - T34	m/z 43.02	12.681	2.41E-03	2.62E+00	1.01E-02	DD - Dj	DD - T34	
m/z 63.045	216.85	2.44E-08	7.6134	4.0899E-07	DD - Dj	T34 - Dj	m/z 92.060	12.172	2.76E-03	2.56E+00	1.10E-02	DD - Dj	DD - T34	T34 - Dj
m/z 91.074	15.026	1.35E-03	2.8683	0.0079333	DD - Dj	T34 - Dj	m/z 105.070	12.11	2.80E-03	2.55E+00	1.10E-02	DD - Dj	DD - T34	
m/z 74.999	14.771	1.44E-03	2.8426	0.0079333	DD - Dj	T34 - Dj	m/z 149.132	11.432	3.38E-03	2.47E+00	1.29E-02	DD - Dj	DD - T34	
m/z 143.106	12.939	2.25E-03	2.6474	0.011	DD - Dj	T34 - Dj	m/z 157.121	10.712	4.16E-03	2.38E+00	1.55E-02	DD - Dj	DD - T34	
m/z 203.180	18.391	6.62E-04	3.1791	0.0067853	DD - T34	Dj - T34	m/z 163.147	10.56	4.36E-03	2.36E+00	1.57E-02	DD - Dj	DD - T34	
m/z 119.085	12.463	2.55E-03	2.5933	0.011569	DD - T34	Dj - T34	m/z 87.044	10.336	4.66E-03	2.33E+00	1.64E-02	DD - Dj	DD - T34	
m/z 107.084	10.142	4.95E-03	2.3057	0.018476	DD - T34	Dj - T34	m/z 111.046	10.058	5.08E-03	2.29E+00	1.74E-02	DD - Dj	DD - T34	

m/z 91.056	9.6095	5.84E-03	2.2333	0.020008	DD - T34	Dj - T34	m/z 129.127	9.9027	5.33E-03	2.27E+00	1.78E-02	DD - Dj	DD - T34	
m/z 123.080	9.5338	5.99E-03	2.2228	0.020008	DD - T34	Dj - T34	m/z 167.056	9.5932	5.87E-03	2.23E+00	1.81E-02	DD - Dj	DD - T34	
m/z 143.144	3063.7	1.77E-13	12.752	2.2504E-11	Dj - DD	Dj - T34	m/z 167.144	9.318	6.42E-03	2.19E+00	1.81E-02	DD - Dj	DD - T34	
m/z 181.159	1081.6	1.90E-11	10.722	8.0294E-10	Dj - DD	Dj - T34	m/z 49.028	8.167	9.49E-03	2.02E+00	2.42E-02	DD - Dj	DD - T34	
m/z 151.148	277.79	8.15E-09	8.0887	1.7258E-07	Dj - DD	Dj - T34	m/z 119.049	7.2804	1.32E-02	1.88E+00	3.23E-02	DD - Dj	DD - T34	
m/z 123.116	17.198	8.43E-04	3.0744	0.0076434	Dj - DD	Dj - T34	m/z 69.069	454.01	9.19E-10	9.04E+00	2.99E-08	DD - Dj	T34 - Dj	
m/z 83.048	15.104	1.33E-03	2.8761	0.0079333	Dj - DD	Dj - T34	m/z 53.013	115.78	3.79E-07	6.42E+00	6.16E-06	DD - Dj	T34 - Dj	
m/z 139.112	13.827	1.80E-03	2.7445	0.0095303	Dj - DD	Dj - T34	m/z 91.056	17.078	8.64E-04	3.06E+00	5.61E-03	DD - Dj	T34 - Dj	
m/z 83.083	13.056	2.19E-03	2.6604	0.011	Dj - DD	Dj - T34	m/z 67.053	15.099	1.33E-03	2.88E+00	7.53E-03	DD - Dj	T34 - Dj	
m/z 167.144	12.694	2.40E-03	2.6197	0.011291	Dj - DD	Dj - T34	m/z 81.034	12.986	2.23E-03	2.65E+00	9.64E-03	DD - Dj	T34 - Dj	
m/z 221.191	10.84	4.01E-03	2.3968	0.015436	Dj - DD	Dj - T34	m/z 53.002	9.4652	6.12E-03	2.21E+00	1.81E-02	DD - Dj	T34 - Dj	
m/z 69.069	9.8789	5.37E-03	2.2703	0.019472	Dj - DD	Dj - T34	m/z 109.102	9.4471	6.16E-03	2.21E+00	1.81E-02	DD - Dj	T34 - Dj	
m/z 193.160	9.681	5.71E-03	2.2432	0.020008	Dj - T34		m/z 42.042	9.3813	6.29E-03	2.20E+00	1.81E-02	DD - Dj	T34 - Dj	
m/z 97.028	609.05	2.48E-10	9.6059	7.8683E-09	T34 - DD	T34 - Dj	m/z 43.055	9.2197	6.63E-03	2.18E+00	1.83E-02	DD - Dj	T34 - Dj	
m/z 107.049	11.465	3.35E-03	2.4747	0.013731	T34 - DD	T34 - Dj	m/z 57.032	9.0326	7.05E-03	2.15E+00	1.91E-02	DD - Dj	T34 - Dj	
							m/z 39.024	8.8139	7.59E-03	2.12E+00	2.01E-02	DD - Dj	T34 - Dj	
							m/z 40.026	8.5439	8.32E-03	2.08E+00	2.16E-02	DD - Dj	T34 - Dj	
							m/z 77.059	616.92	2.34E-10	9.63E+00	1.01E-08	DD - T34	Dj - T34	
							m/z 91.074	17.198	8.43E-04	3.07E+00	5.61E-03	DD - T34	Dj - T34	
							m/z 87.080	9.7886	5.52E-03	2.26E+00	1.79E-02	DD - T34	Dj - T34	
							m/z 59.051	9.4182	6.21E-03	2.21E+00	1.81E-02	DD - T34		
							m/z 83.048	7.5733	1.18E-02	1.93E+00	2.95E-02	DD - T34		
							m/z 103.076	19.511	5.34E-04	3.27E+00	5.26E-03	Dj - DD	Dj - T34	
							m/z 155.146	13.863	1.79E-03	2.75E+00	8.93E-03	Dj - DD	Dj - T34	
							m/z 119.085	13.69	1.86E-03	2.73E+00	8.97E-03	Dj - DD	Dj - T34	
