# Supplementary figures and images for: Trichoderma harzianum Volatile Organic Compounds Regulated by the THCTF1 Transcription Factor Are Involved in Antifungal Activity and Beneficial Plant Responses
Source: J Fungi (Basel). 2023 Jun 11;9(6):654. doi: 10.3390/jof9060654 (PMC10302578; doi:10.3390/jof9060654)

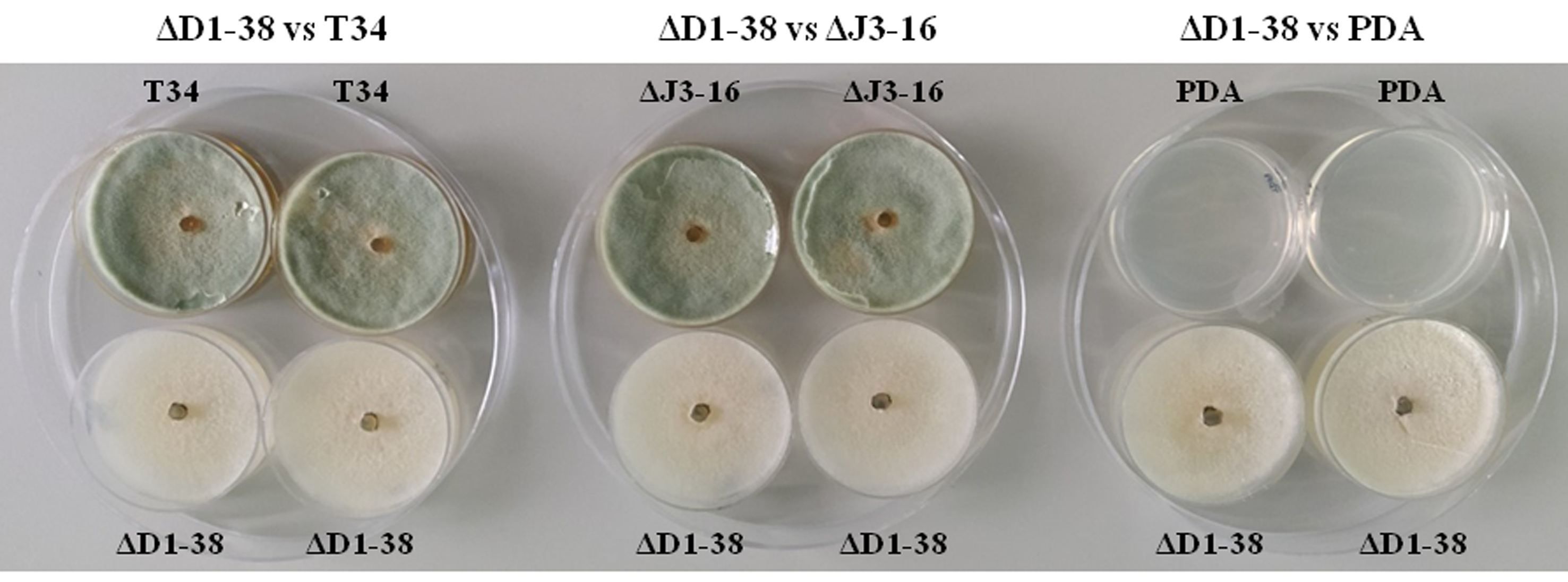

Supplement: Supplementary file 1 [file jof-09-00654-s001.zip › Figure S1.tif]

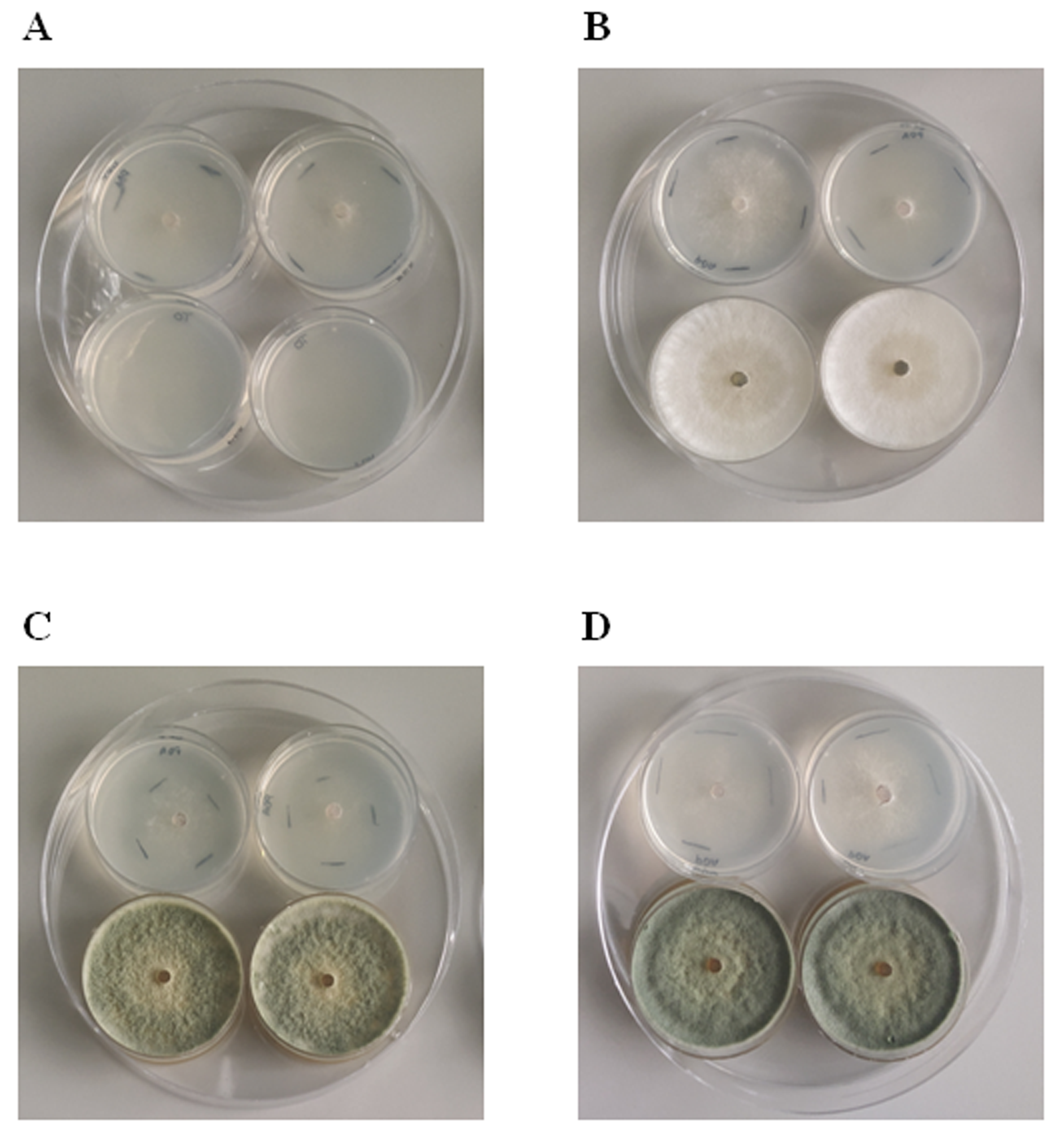

Supplement: Supplementary file 1 [file jof-09-00654-s001.zip › Figure S2.tif]
